# Seed size effects on plant establishment under low atmospheric CO_2_, with implications for seed size evolution

**DOI:** 10.1093/aob/mcac112

**Published:** 2022-09-12

**Authors:** Honour C McCann, Rowan F Sage

**Affiliations:** Department of Ecology and Evolutionary Biology, University of Toronto, Toronto, ON, M5S 3B2, Canada; Max Planck Institute for Biology, Tübingen, Germany; Department of Ecology and Evolutionary Biology, University of Toronto, Toronto, ON, M5S 3B2, Canada

**Keywords:** Atmospheric CO_2_, global change, seed ecology, seed evolution, seedling establishment, seedling mortality

## Abstract

**Background and Aims:**

Low atmospheric CO_2_ concentration depresses photosynthesis and resource use efficiency, and therefore can inhibit phases of the life cycle such as seedling establishment. Seed reserves can compensate for photosynthetic inhibition by accelerating seedling growth. We therefore hypothesize that seedlings arising from large seeds show less inhibition from low atmospheric CO_2_ than young plants from small seeds. Seed size effects on seedling responses to low CO_2_ may also be enhanced in warm environments, due to greater photorespiration at high temperature.

**Methods:**

*Phaseolus* and *Vigna* seeds differing in mass by over two orders of magnitude were planted and grown for 14 d in growth chambers with CO_2_ concentrations of 370, 180 or 100 ppm, in thermal regimes of 25 °C/19 °C, 30 °C/24 °C or 35 °C/29 °C (day/night). We measured leaf area expansion, shoot growth and mortality of the seedlings arising from the variously sized seeds at 14 days after planting (14 DAP).

**Key Results:**

Relative to small-seeded plants, large-seeded genotypes produced greater leaf area and shoot mass at 14 DAP across the range of CO_2_ treatments in the 25 °C/19 °C and 30 °C/24 °C regimes, and at 100 ppm in the 35 °C/29 °C treatment. The proportional decline in leaf area and seed mass with CO_2_ reduction was generally greater for seedlings arising from small than from large seeds. Reductions in leaf area due to CO_2_ reduction increased in the warmer temperature treatments. In the 35 °C/19 °C treatment at 100 ppm CO_2_, seedling mortality was greater in small- than in large-seeded genotypes, and the small-seeded genotypes were unable to exit the seedling stage by the end of the experiment.

**Conclusions:**

The results support a hypothesis that seedlings from large seeds grow and establish better than seedlings from small seeds in warm, low CO_2_ environments. During low CO_2_ episodes in Earth’s history, such as the past 30 million years, large seeds may have been favoured by natural selection in warm environments. With the recent rise in atmospheric CO_2_ due to human activities, trade-offs between seed size and number may already be affected, such that seed size today may be non-optimal in their natural habitats.

## INTRODUCTION

Atmospheric CO_2_ concentration has varied substantially over Earth’s history (Berner, 2006). Between 200 and 40 million years ago (mya), it was often two or more times higher than today’s value of 418 ppm but declined in the past 35 mya to approach values as low as 180 ppm some 20 000 year ago (Supplementary data Fig. S1; Lüthi *et al.*, 2008; Zhang *et al.*, 2013). Ice core records demonstrate that atmospheric CO_2_ oscillated between 180 and 300 ppm over the past 800 000 years, with most (73 %) of this period spent below 250 ppm (Supplementary data Fig. S1; Lüthi *et al.*, 2008). Hence, low atmospheric CO_2_ has prevailed long enough to have allowed Earth’s flora to evolve low CO_2_ adaptations (Sage and Coleman, 2001). Possible low CO_2_ adaptations include higher CO_2_ specificity of the CO_2_-fixing enzyme Rubisco, greater stomatal density, carbon-concentrating mechanism such as C_4_ photosynthesis and, possibly, larger seed mass to offset CO_2_ limitations on establishment and early seedling growth (Ehleringer *et al.* 1991; Sage and Coleman, 2001; Campbell *et al.*, 2005; Zhu *et al.*, 2007; Gerhart and Ward, 2010; Sage *et al.*, 2018). If the modern flora is adapted to low atmospheric CO_2_, it is possible that these adaptations constrain responses to rising CO_2_, such that plants today might already be maladapted following recent anthropogenic CO_2_ enrichment (Sage and Coleman, 2001).

Plant responses to elevated atmospheric CO_2_ above recent values are well studied (reviewed in Ainsworth and Long, 2005; Leakey *et al.*, 2012; Poorter *et al.*, 2022); however, responses to low atmospheric CO_2_ have received much less attention (reviewed in Gerhart and Ward, 2010). It is well recognized that atmospheric CO_2_ enrichment acts like a fertilizer to differentially accelerate seedling growth and establishment, potentially to such a degree that ecosystems will experience turnover in community assemblages (Davis *et al.*, 2007; Bond and Midgley, 2012; Polley *et al.*, 2013; Poorter *et al.*, 2022). In contrast, low CO_2_ relative to the current ambient level reduces growth, reproduction and fitness by slowing photosynthetic CO_2_ assimilation and inhibiting efficiencies of water, nitrogen and light use (Sage, 1995; Tissue *et al.*, 1995; Polley *et al.*, 2003; Campbell *et al.*, 2005; Campbell and Sage, 2006; Gerhart and Ward, 2010). Of particular note, low CO_2_ limitations are aggravated in warmer climates, because the inhibitory process of photorespiration is enhanced by combinations of low CO_2_ and high temperatures (Ehleringer *et al.*, 1991; Santrucek and Sage, 1996; Sage, 2013). Seedlings may be particularly prone to photosynthesis and growth inhibition caused by low CO_2_, because they must grow through the surface boundary layer where solar heating enhances leaf temperature and thus would aggravate photorespiration. Small seedlings, in particular, may lack sufficient leaf area to compensate for direct inhibition of photosynthesis by CO_2_ deficiency, particularly since early growth of plants is directly related to the rate of leaf area expansion (Monteith, 1977; Potter and Jones, 1977; Gifford and Evans, 1981). In tobacco, for example, seedlings grown at low CO_2_ (100 and 150 ppm) and warm temperatures (30 °C) were slow to exit the seedling stage rather than rapidly entering exponential growth and establishing, as observed for seedlings grown at higher CO_2_ (Campbell *et al.*, 2005). Campbell *et al.* (2005) observed that once tobacco seedlings in low CO_2_ produced a few centimetres of leaf area, they were able to enter exponential growth, establish and eventually reproduce in the same low CO_2_ and warm conditions that delayed their exiting the seedling stage. A similar delay of seedling growth in low CO_2_ has been observed in beans (*Phaseolus vulgaris* grown at 200 ppm CO_2_ and 36 °C/29 °C day/night temperature; Cowling and Sage, 1998) and *Abutilon theophrastii* (at 150 ppm and 28 °C/22 °C; Dippery *et al.*, 1995). Because arrested seedlings are more vulnerable to chance mortality and competitive suppression, Campbell *et al.* (2005) hypothesized that strong selection pressure against plants stuck in the seedling stage by low CO_2_ could favour compensatory adaptations, most notably increased seed size that provides greater carbon reserves to support early growth and establishment. This hypothesis was informed by trends towards larger seeds observed in modern plants adapted to environmental hazards such as drought and shade that reduce establishment success (Leishman *et al.*, 2000; Westoby *et al.*, 2002). Mechanistically, larger seeds enhance the initial reach of shoots and roots, thereby reducing the time required for seedlings to become photosynthetically independent, homeohydric and nutrient sufficient (Raven, 1999; Leishmann *et al.*, 2000; Benard and Toft, 2007). Low atmospheric CO_2_ can also be considered a significant environmental hazard, particularly when coupled with other stresses such as shade, drought and heat (Sage, 1995; Cowling and Sage, 1998). No study, however, has considered interactions between early growth, establishment potential and low atmospheric CO_2_ availability between plants of varying seed size.

Here, we examine the hypothesis that relative to current atmospheric CO_2_ concentration, larger seeds enable proportionally greater seedling growth in low CO_2_ atmospheres than smaller seeds, particularly where low CO_2_ and elevated temperatures accelerate photorespiration. Where low CO_2_ is particularly inhibitory, larger seed reserves may enable growth and establishment in conditions where plants arising from small seeds fail to establish. To test our hypotheses, we examined seedling growth and mortality in the first 2 weeks after germination in five bean species whose seed weights vary by 2.5 orders of magnitude. Seedings were grown in plant growth chambers at three CO_2_ concentrations (100, 180 and 380 ppm) and three thermal regimes (25 °C/19 °C, 30 °C/24 °C and 35 °C/29 °C day/night temperature). We selected 2 weeks after planting as the experimental period because this duration allows bean seedlings to become autotrophic and enter exponential growth. The experiment thus focuses on seed size effects on growth and survival through the establishment window.

## MATERIALS AND METHODS

### Study species

**Fig. 1.**
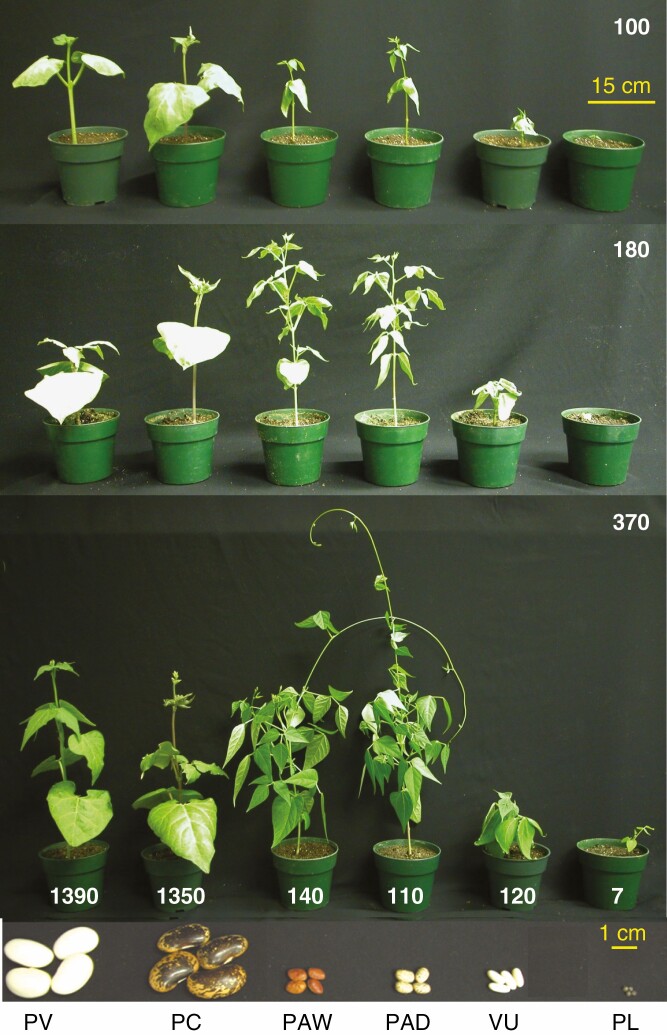
Growth form, and plant and seed sizes of bean genotypes used in this study. Treatment conditions were 100, 180 or 370 ppm growth CO_2_ and 35 °C/29 °C day/night temperature. Seed size in milligrams is written over each pot. From left to right: *Phaseolus vulgaris* ‘Pueblo’ (PV); *P. coccineus* ‘Painted Lady’ scarlet runner (PC); wild *P. acutifolius* (PAW); domesticated *P. acutifolius* (‘Brown Tepary’, PAD); *Vigna umbellata* (VU); and *Phaseolus leptostachyus* (PL). The photo was taken at 14 DAP.

Five genotypes from four *Phaseolus* species, and one rice bean (*Vigna umbellata*) genotype were selected for the study (Fig. 1; Supplementary data Table S1). *Vigna* is closely related to *Phaseolus* and shares similar growth form and habitat (annual life cycle, climbing tendrils, warm season and ruderal habitats; Goel *et al.*, 2002). The large-seeded species in the study are *Phaseolus vulgaris* (‘Pueblo’) and *P. coccineus* (‘Painted Lady’ scarlet runner bean), which have average dry seed weights of 1.35 and 1.39 g, respectively (Supplementary data Table S1). Smaller seeded species are the rice bean *Vigna umbellata* (seed weight = 0.12 g), domesticated (‘Brown Tepary’, 0.11 g) and non-domesticated (0.14 g) *P. acutifolius* and non-domesticated *P. leptostachyus* (0.007 g). Seeds of *P. vulgaris*, *P. coccineus*, *P. acutifolius* ‘Brown Tepary’ and *Vigna umbellata* were procured from Phipps Country store (Pescadaro, CA, USA; no longer in business; Supplementary data Table S1). Seeds of non-domesticated *P. acutifolius* and *P. leptostachyus* were obtained from the USDA Western Regional Plant Introduction Station (Pullman, WA, USA).

### Growth conditions and treatments

Seeds were planted in 1 L pots filled with soil (Sunshine Germination Mix #3, Sun Gro Horticulture Inc., Bellevue, WA, USA; Fig. 1). Seedlings were watered and fertilized daily with a 0.25 strength Hoagland’s solution (Epstein and Bloom, 2005). Seedlings were grown in controlled-environment chambers (Biochambers Model GC-20, Winnipeg, MB, Canada) under a 12 h photoperiod at a mean photosynthetic photon flux density of 550 μmol photons m^–2^ s^–1^ at the top of the leaf canopy. Germination occurred in the chambers under experimental treatment conditions. Treatment day/night temperatures were 25 °C/19 °C, 30 °C/24 °C and 35 °C/29 °C, which were selected to represent typical sub-optimal, optimal and supra-optimal thermal conditions in growth environments of beans (Bitocchi *et al.*, 2017). Growth CO_2_ concentrations were 100, 180 and 370 ± 10 ppm CO_2_. The lowest CO_2_ treatment (100 ppm) was selected to aggravate low CO_2_ effects that may be apparent at 180 ppm; the 180 ppm treatment corresponds to the approximate lowest CO_2_ concentration recorded in polar ice core records over the past 800 000 years (Lüthi *et al.*, 2008). The 370 ppm treatment corresponds to CO_2_ concentrations at the turn of the millennium (CO2now.org). The chamber CO_2_ concentration was controlled using a CO_2_ gas analyser (Model WMA-2, 3 or 4; PP Systems, Haverhill, MA, USA) which regulated the removal of CO_2_ by circulating air through a soda-lime scrubber (Campbell *et al.*, 2005). Seedlings were harvested 14 days after planting (DAP) and oven-dried at 70 °C. Mortality at 14 d after germination was assessed as the number of seedlings that died following successful germination in the 14 d growth period. Leaf area was measured immediately following harvest using a Li-Cor leaf area meter (LI-3000, Li-Cor, Lincoln, NE, USA). Samples were then dried and shoot biomass measured.

### Experimental design and statistical analysis

For all species except *P. leptostachyus*, wild *P. acutifolia* and *V. umbellata* at 35 °C, the nine treatment combinations (three temperatures × three CO_2_ levels) were replicated twice in the growth response study and once in the mortality assessment. Replicate treatments were switched between plant growth chambers. Seed number limitations allowed for only one replication of tiny-seeded *P. leptostachyus* at just the 35 °C/29 °C treatment. Sample size within each treatment replication and genotype was initially 15, although mortality reduced the final sample number at the time of harvest as indicated in figure 6. Statistical analysis was carried out using R [three-way analysis of variance (ANOVA); https://www.r-project.org] or SigmaPlot Version 14.5 (one-way ANOVA and regression analysis; Inpixon, Palo Alto, CA, USA) with *P < *0.05 as the critical level of significance. After square-root transformation to establish normality in the leaf area and shoot mass data, the results were evaluated with a three-way ANOVA using R. The data were then re-evaluated with one-way ANOVA and/or linear regression analysis (both with Sigmaplot) to evaluate differences between specific CO_2_, temperature or seed size groups. Where data were non-normal in the one-way ANOVA tests, differences were evaluated on ranks, and a Dunn’s post-hoc test was used to determine significantly different treatment and genotype groups. Where a normal distribution was apparent, a Tukey’s post-hoc test was used to evaluate differences between individual groups.

## RESULTS

### Leaf area and biomass production

Figure 1 shows representative plants from the 35 °C/29 °C treatment grown at 100, 180 and 370 ppm CO_2_ concentrations at 14 DAP. Pot size, seed size, seed mass and plant size are shown for the three CO_2_ treatments. A strong CO_2_ effect on shoot mass and leaf area is evident in the photos, with plants being smaller in the lower CO_2_ treatments. In the two low CO_2_ treatments, the tiny-seeded *P. leptostachyus* failed to produce enough biomass to grow above the lip of the pot. In contrast, the two large-seeded varieties produced large primary leaves that dominated the early leaf area, particularly at low CO_2_.

**Fig. 2.**
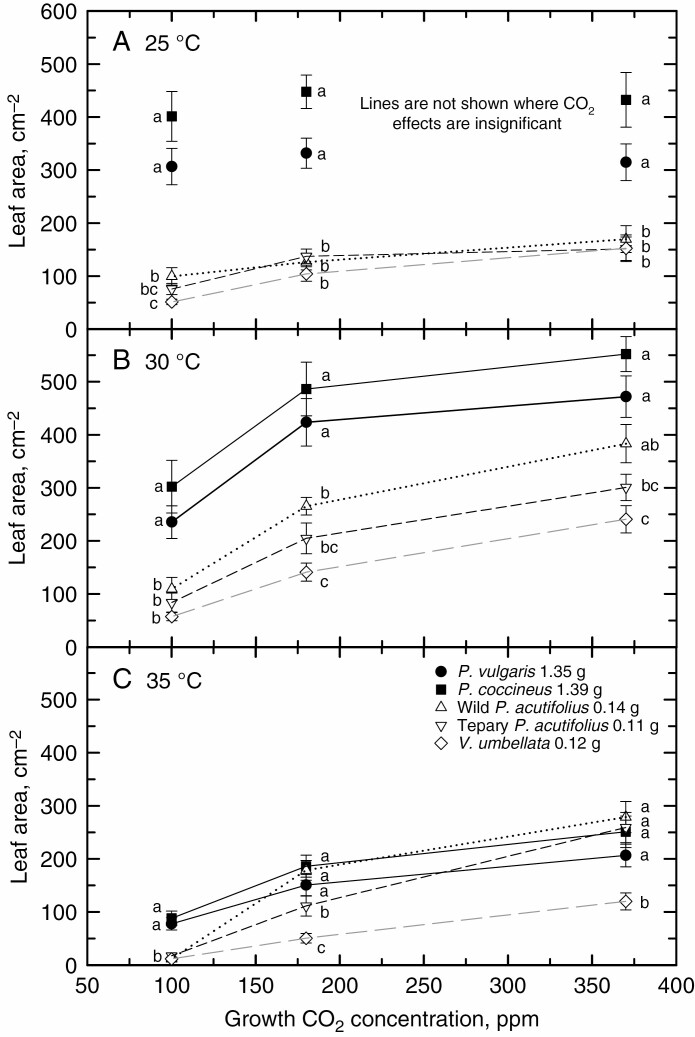
Leaf area at 14 DAP for five bean varieties grown at three ambient CO_2_ concentrations (100, 180 and 370 ppm) at day/night temperatures of either 25 °C/19 °C (A), 30 °C/24 °C (B) or 35°C /29 °C (C). Plant variety and seed weight corresponding to each symbol are indicated in (C). Means ± 95 % confidence interval, *n* = 15–30 for each symbol, using data from both replicates. Lines connecting the data are only shown for responses where CO_2_ effects are significant by both one-way ANOVA and linear regression analysis at *P* < 0.05. Letters next to symbols indicate significantly distinct genotype groups within a CO_2_ treatment at *P* < 0.05. See Supplementary data Table S2 for a three-way ANOVA table for CO_2_, temperature and seed size treatment effects, and their interactions. See Supplementary data Fig. S2 for the corresponding dry weight responses to CO_2_ and temperature.

We first examine a hypothesis that differences in leaf area at 14 DAP between plants arising from small vs. large seeds are relatively greater in low relative to current growth CO_2_ concentrations. Across the CO_2_ treatments, the large-seeded genotypes (*P. vulgaris* and *P. coccinea*) produced 2–5 times more leaf area at 14 DAP than the small-seeded genotypes in the 25 °C/19 °C treatment (Fig. 2A). In this thermal regime, CO_2_ effects on leaf area were absent in the large-seeded genotypes. In the small-seeded genotypes, in contrast, less leaf area was produced in the 100 ppm compared with the 370 ppm CO_2_ treatment (Fig. 2A). In the 30 °C/24 °C condition at 370 ppm CO_2_, leaf area was greater in all species than observed under the 25 °C/19 °C daytime temperature at 370 ppm, reflecting the warm thermal optimum generally observed for bean species (Bitocchi *et al.*, 2017). At 100 ppm CO_2_ and 30 °C/24 °C, the large-seeded plants produced significantly less leaf area than at 370 ppm, in contrast to the insignificant CO_2_ response observed at 25 °C/19 °C; however, in the small-seeded plants, a proportionally larger reduction in leaf area was observed with CO_2_ reduction at this temperature (Fig. 2B). At 35 °C daytime temperature, leaf area production declined in all species relative to the 30 °C growth treatment (Fig. 2C). The growth reduction at 380 ppm was more severe in the large-seeded species than in the small-seeded tepary bean lines (*P. acutifolius* varieties), reflecting greater heat tolerance of tepary beans (Bitocchi *et al.*, 2017). However, at 100 ppm CO_2_, leaf area production collapsed in each small-seeded line, including the heat-tolerant tepary lines. In contrast, leaf area collapse was not observed in the large-seeded lines, which were able to produce 80–100 cm^2^ of leaf area at 14 DAP in the 35 °C treatment at 100 ppm.

**Fig. 3.**
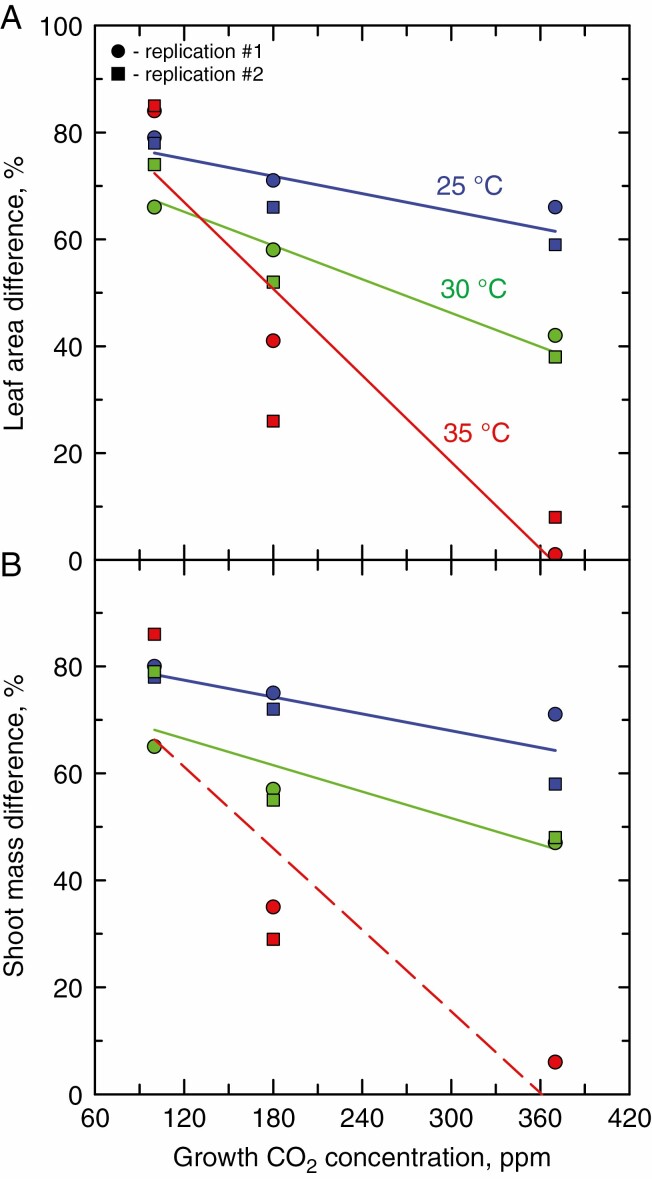
The percentage difference at 14 DAP between large- and small-seeded plants of (A) leaf area and (B) shoot mass. Values were calculated by pooling all observations in a single experimental replicate at the indicated CO_2_ and temperature levels, first for large-seeded species (*P. vulgaris* and *P. coccineus*) and then for small-seeded species (wild *P. acutifolia*, domesticated *P. acutifolia* and *V. umbellata*). The tiny-seeded *P. leptostachyus* is absent from this analysis. Percentage differences were calculated as [100 % × (large seeded means – small seeded means)/large seeded means]. All regression slopes are significant at *P* < 0.05, except for shoot mass differences at 35 °C (dashed line, slope *P* = 0.14). In the 35 °C/29 °C treatment, the absence of the wild *P. acutifolius* trial in replicate 2 at 370 ppm, and the *V. umbellata* trial in replicate 1 at 100 ppm reduced the power of the shoot mass regression to resolve significant trends.

**Fig. 4.**
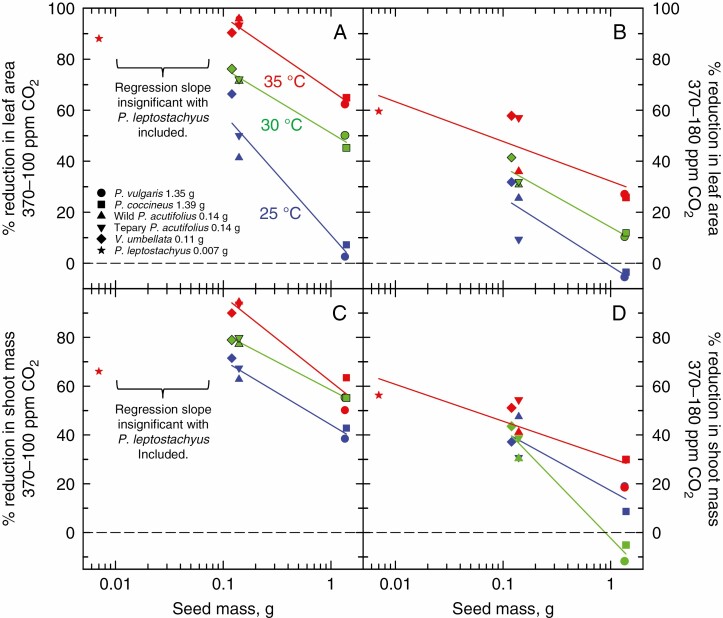
The percentage reduction in leaf area (A, B) and shoot mass (C, D) for large- (seed mass 1.38 ± 0.02 g) and small-seeded bean genotypes (seed mass near 0.13 ± 0.02 g or 0.007 g) grown at low CO_2_ concentration in comparison with values at 370 ppm CO_2_ for day/night temperature treatments of 25 °C/19 °C (blue lines and symbols), 30 °C/24 °C (green lines and symbols) or 35 °C/29 °C (red lines and symbols). The symbol legend in red font applies to all three temperature regimes. Low CO_2_ treatments were 100 ppm CO_2_ (A and B) or 180 ppm (C and D). Mean ± s.d., *n* = 15–30 using data from both replicates. See Fig. 2 for original leaf area means and Supplementary data Fig. S2 for the original shoot mass means. All regression lines shown have significant slopes at *P* ≤ 0.05. In (A) and (C), regressions at 35 °C that include *P. leptostachyus* were insignificant and are not shown.

A three-way ANOVA using all species except *P. leptosytachyus* identified significant CO_2_, temperature and seed size effects on leaf area at 14 DAP, and significant interactions, notably between CO_2_ and seed size, and CO_2_ plus temperature and seed size (Supplementary data Table S2). To better show the interaction between seed size and CO_2_, we plotted the relative differences in leaf area produced by small- and large-seeded plants at 14 DAP as a function of growth CO_2_. At each temperature, the percentage difference in leaf area between small- and large-seeded plants increased at the lower CO_2_ concentrations, particularly in the warmer growth treatments (Fig. 3A). We then examined the relative reduction in leaf area caused by declining growth CO_2_ as a function of seed mass (Fig. 4A, B). We observed a negligible reduction in leaf area at 25 °C/19 °C in the large-seeded species when CO_2_ was reduced from 370 ppm to either 100 or 180 ppm. In contrast, in the small-seeded species at 25 °C/19 °C, the reduction in CO_2_ from 370 to 100 ppm reduced the leaf area of small-seeded species by >40 %. The reduction of CO_2_ from 370 to 180 ppm reduced leaf area by close to 20 % on average. Under the warmer growth regimes, the reduction in leaf area with CO_2_ reduction increased in all species, but to a greater degree in small- than in large-seeded species. On average, reduction of CO_2_ from 370 to 100 ppm resulted in a 50 % (at 30 °C daytime temperature) to 63 % (at 35 °C) reduction in leaf area in the large-seeded species, and a 73 % (at 30 °C) to >88 % (at 35 °C) reduction of leaf area in the small-seeded species (Fig. 3A). Leaf area decline with a reduction in CO_2_ from 370 to 180 ppm was less in magnitude than observed between 370 and 100 ppm, but showed a similar pattern of change in that at warmer temperatures, the degree of leaf area reduction was greater in the small- than in the large-seeded species (Fig. 4B).

At each temperature, the difference in leaf weight between small- and large-seeded plants also increased at the lower CO_2_ concentrations, particularly in the warmer growth treatments (Fig. 3B; Supplementary data Fig. S2). The reduction in shoot mass with reduction in growth CO_2_ was generally greater in the small- than in the large-seeded genotypes (Fig. 4C, D; Supplementary data Fig. S2). Warming temperatures aggravated the low CO_2_ inhibition of biomass at 100 ppm, where the small-seeded *P. acutifolius* and *V. umbellata* plants grown at 35 °C exhibited on average a >90 % decline in biomass relative to their counterparts grown at 370 ppm (Fig. 4C). By comparison, biomass decline in the large-seeded plants between 370 and 100 ppm was about 60 % at 35 °C (Fig. 4C).

In the case of *P leptostachyus* at 35 °C, the relative reduction in leaf area at 100 ppm relative to 370 ppm was similar to that in the small-seeded *P. acutifolius* and *V. umbellata* lines (Fig. 4C), while the relative reduction in seed mass of *P. leptostachyus* was similar to that of the large-seeded lines. The result was a loss of significance in the regression of seed mass vs. relative CO_2_ effect at 35 °C when *P. leptostachyus* was included in the regression (Fig. 4A, C).

### *CO*_*2*_ *effects on growth in* P. leptostachyus

**Fig. 5.**
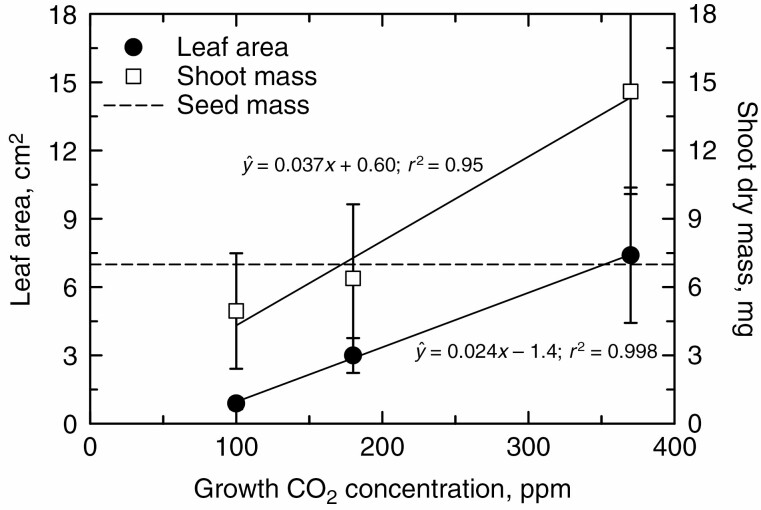
The response of leaf area and shoot dry mass to growth CO_2_ treatment in the small-seeded *Phaseolus leptostachyus* at 14 DAP and a day/night temperature of 35 °C/29 °C. The mean seed weight of the genotype (0.007 g) is shown as a dashed line. Mean ± s.d. Linear regressions with significant slopes at *P* < 0.05 are shown with their corresponding equations.

We hypothesized that bean plants arising from tiny seeds would be particularly inhibited in their early growth by low CO_2_, and would become stuck in the seedling stage, particularly in the low CO_2_ and warm treatments. To test this hypothesis, we grew *P. leptostachyus*, a twining wild bean species arising from seeds weighing around 7 mg. Due to seed availability limitation, we only grew *P. leptostachyus* at 35 °C/29 °C day/night temperature. A strong CO_2_ effect was observed at 14 DAP, with leaf area being lower by >50 % in the 180 ppm compared with the 370 ppm treatment, and lower by 88 % in the 100 ppm compared with the 370 ppm treatment (Figs 4A, B and 5). Shoot biomass also showed a large inhibition following CO_2_ reduction, with a 59 % decline in biomass from 370 to 180 ppm, and a 66 % decline from 370 to 100 ppm (Figs 4C, D and 5). Notably, the shoot mass was less than the seed mass in the 100 ppm treatment (Fig. 5). If we assume that root mass is 40 % of shoot mass (a typical ratio in unstressed beans; Berny-Mier y Teran *et al.*, 2019), the mass of the *P. leptostachyus* plants (about 6.5 mg) under the 100 ppm treatment would be less than its seed mass of 7 mg, demonstrating that growth is nil in this treatment. Given this negligible mass increment, it is probable that residual seed mass contributed to the final mass of the *P. leptostachyus* plants, lowering the apparent effect of CO_2_ reduction on biomass to 66 %, below the approx. 90 % reduction observed between the 370 and 100 ppm treatments in the *P. acutifolius* and *V. umbellata* lines at 35 °C. Assuming the same root mass allocation, the whole-plant mass increment at 180 ppm would yield just 40 % more biomass than seed mass, leading to the tiny plants seen in Fig. 1.

### Seedling mortality

**Fig. 6.**
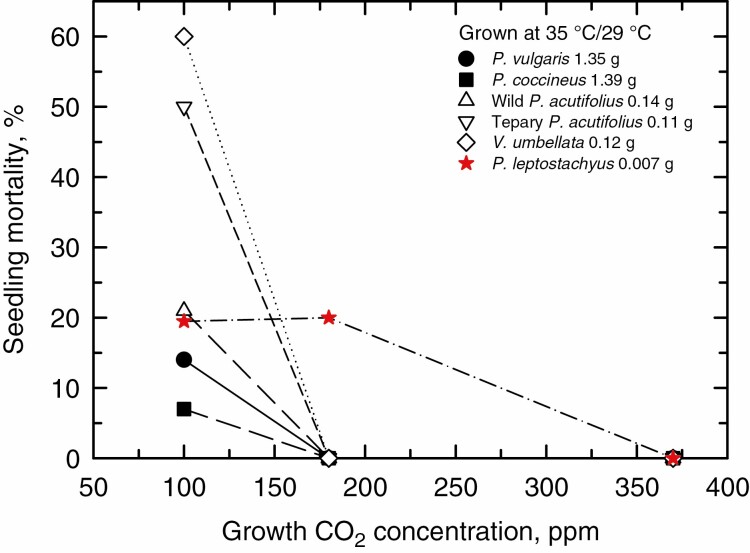
The effect of growth CO_2_ on percentage seedling mortality at 14 DAP for seedlings grown at 35 °C/29 °C day/night temperature. Mortality in the 30 °C/24 °C and 25 °C/19 °C treatments was minor to nil under each CO_2_ treatment, being below 7 % (not shown).

Mortality of germinated seedlings at 14 DAP was minor (>90 % survivorship) in all genotypes and CO_2_ levels in the 25 °C/19 °C and 30 °C/24 °C day/night treatments (data not shown). In the 35 °C/29 °C growth regime, seedling mortality was nil at 370 and 180 ppm for all genotypes except the tiny-seeded *P. leptostachyus*, where 20 % of the seedlings died at 180 ppm (Fig. 6). At 100 ppm, ≤14 % of large-seeded genotypes died by 14 DAP, while 20–60 % of the small-seeded genotypes died.

## DISCUSSION

These results support the hypothesis that seedlings germinating from larger seeds are less inhibited by low CO_2_ conditions than seedlings arising from smaller seeds, particularly at warmer temperatures when photorespiration is enhanced. We observed that small-seeded species have a greater proportional reduction in leaf area and shoot mass with CO_2_ reduction than large-seeded bean plants. In particular, small-seeded species became arrested at the seedling stage in the lowest CO_2_ treatments in the 35 °C/29 °C temperature regime, while the large-seeded species showed sustained growth. Coupled with this slow growth was generally higher seedling mortality at 100 ppm for the small-seeded species compared with seedlings produced by large seeds. Mechanistically, we attribute some of the results to the observation that the large-seeded species produced large primary leaves immediately after germination. We hypothesize that the large leaves offset photosynthetic limitations at low CO_2_, allowing the plants to acquire sufficient carbon to sustain exponential growth and enter a condition of autotrophy, homeohydry and nutrient sufficiency, which are the hallmarks of successful establishment (Raven, 1999; Campbell and Sage, 2005). Without this large initial leaf area produced using large seed reserves, the small-seeded species exhibited greater sensitivity to CO_2_ reduction, particularly at elevated temperature. In the smallest seeded *P. leptostachyus* plants at 35 °C/29 °C, the situation was so severe that the plants grown at low CO_2_ exhibited little net biomass accumulation relative to the original seed mass.

Our study examined leaf and shoot growth in environments of sufficient water and nutrient supply, and did not include realistic hazards such as disease, herbivory, shade, drought and fire that aggravate mortality of seedlings during establishment (Westoby *et al.*, 2002). Despite this lack of hazards other than heat and low CO_2_, we still observed chance mortality in the 100 ppm CO_2_ treatment at 35 °C/29 °C, but more so in the small-seeded species, including the heat-adapted *P. acutifolius* lines. Storage reserves in general are recognized as buffers against environmental stress because they allow plants to direct internal resources towards traits that alleviate the associated hazard, for example more to leaf area in low CO_2_ or shade, and more to root mass in dry soils (Chapin *et al.*, 1990; McPherson and Williams, 1998; Benard and Toft, 2007; Myers and Kitijima, 2007). We therefore hypothesize that seedling mortality would be even greater in realistic environments where hazards can combine with low CO_2_ to enhance mortality in poorly resourced plants arising from small seeds. While the 100 ppm CO_2_ treatment lacks a natural precedent; it indicates how prehistoric CO_2_ environments in the 180–250 ppm range could have promoted mortality if coupled with other stresses that also impair carbon gain. We also note that our results pertain to relatively short-lived annuals and perennials differing in seed size, where mortality during establishment would have a direct impact on subsequent population size and fecundity. Many larger seeded species are long-lived perennials such as forest trees that are subject to mortality over multiple years before reaching reproductive maturity; hence the fitness consequences of seed size variation during establishment can be diminished (Moles *et al.*, 2005*a*, *b*; Moles, 2018). In these species, larger seed size can sustain seedlings longer while waiting for favourable establishment conditions, such as the opening of canopy gaps in forest interiors (Kidson and Westoby, 2000; Leishman *et al.*, 2000). Low CO_2_ would constrain the boundaries of what would constitute a favourable condition, for example by requiring a larger gap size to provide enough carbon for establishment. Thus, while seed size consequences for fitness may weaken through time in long-lived species, we hypothesize that low CO_2_ effects are still important, though they may influence aspects of seedling performance different from those in short-lived species.

In the course of low CO_2_ episodes during the Pleistocene Ice Age, air temperatures at low latitudes are modelled to have been 1–6 °C cooler on average than today, but still would have been warm, with summer temperatures exceeding 30 °C (Bonan, 2002; Seltzer *et al.*, 2021). Air temperatures of 35 °C were less common in tropical and sub-tropical environments of the time, raising the question of whether our 35 °C/29 °C treatment has relevance. We believe it does. The temperature of importance for seedling establishment is not the air temperature above the vegetation, but the temperature in the microclimate of the regeneration niche near the soil surface. Here, the surface boundary layer will trap solar radiation while convective and latent cooling of leaves could be reduced by the still air in the boundary layer; hence, seedling temperatures warm by more than a few degrees relative to bulk air temperature (Nobel, 1984; Oke, 1987; Sage and Sage, 2002; Larcher, 2003; Wehner and Wehner, 2011). Daytime air temperatures near 30 °C during the Pleistocene would have corresponded to warmer conditions in the regeneration niche on sunny days, possibly reaching 35 °C. We thus conclude that our hot treatment is a realistic representation of the regeneration environments seedlings faced in the low CO_2_ conditions of recent geological time. In low CO_2_ atmospheres, boundary layer heating would have been more impactful for seedlings arising from small seeds close to the soil surface than for seedlings from large seeds where stored reserves could support rapid growth through the boundary layer to cooler air layers above.

Our study involved beans, a typical C_3_ group of species that originated in lower latitudes (Bitocchi *et al.*, 2017). Our criteria for selecting beans as a study system was their tolerance of warm temperatures in addition to their broad variation in seed size. The natural heat tolerance reduces inhibitory heat stress that could otherwise obscure effects of low CO_2_ in the warmer treatments. The two *P. acutifolius* lines used here are noted for particularly high heat tolerance, more so than other cultivated *Phaseolus* species (Bitocchi *et al.*, 2017). We observed that heat reduced growth of *P. vulgaris*, *P. coccinea* and *Vigna* species at 35 °C to a greater extent than in the two *P. acutifolius* lines, such that the *P. acutifolius* genotypes produced similar leaf area and shoot mass to the large-seeded *P. vulgaris* and *P. coccineus* genotypes at 370 ppm. Despite this, at 100 ppm, all the small-seeded genotypes including the *P. acutifolius* lines showed a marked reduction in growth compared with the large-seeded genotypes of *P. vulgaris* and *P. coccinea*.

### Implications for seed size evolution

The contribution of seed size to plant fitness has received much attention in recent years (Leishman *et al.*, 2000; Moles *et al.*, 2005*a*, *b*; Myers and Kitijima, 2007; Eriksson, 2008; Sims, 2012; Moles, 2018; Martin, 2021). Seed size is influenced by multiple environmental variables, including water and nutrient availability, dispersal mechanisms, growth form and life history characteristics, such that a trade-off can exist between seed size and number (Leishmann *et al.*, 2000; Moles and Westoby, 2004; Moles *et al.*, 2005*a*, *b*; Moles, 2018). At near-current CO_2_ levels of recent history, production of many smaller seeds enhances fecundity and the probability of establishment in sites with favourable conditions, while larger seeds enhance initial establishment success, particularly in non-optimal environments (Leishmann *et al.*, 2000; Westoby *et al.*, 2002; Moles and Westoby 2004; Muller-Landau, 2010). In favourable environments, however, investing in fewer, large seeds can reduce fecundity and colonization opportunities in plants of similar life history (Turnbull *et al.*, 1999; Moles and Westoby, 2004). If seedling growth, survivorship and establishment are hindered in warm, low CO_2_ environments to a greater degree in small- than large-seeded species, then we hypothesize that natural selection would favour larger seeds in warm atmospheres of low CO_2_. We recognize that other factors will also influence seed size evolution and could potentially over-ride influences of CO_2_; however, low CO_2_ has not been considered as a factor influencing seed size evolution in the modern flora, so legacies of any CO_2_ effects are unknown. Future studies of seed size trade-offs might consider such possibilities.

Circumstantial evidence in the paleontological record is consistent with a hypothesis that CO_2_ influences seed size evolution. For example, angiosperm seed sizes in the lower CO_2_ epochs of the past 50 million years are generally larger than during higher CO_2_ episodes of the Cretaceous period before 70 mya (Tiffney, 1984; Eriksson, 2008). In addition, seed size of ancient gymnosperms increased during the Mississippian period between 360 and 320 mya, a time modelled to coincide with atmospheric CO_2_ decline (Berner, 2006; Sims, 2012). Seed sizes in the modern flora are larger on average in warmer, low latitude environments than higher, cooler latitudes (Moles *et al.*, 2004, 2007), consistent with the observation that higher rates of photorespiration in warm environments would have aggravated low CO_2_ limitations and potentially hindered seedling success (Cowling and Sage, 1998; Campbell *et al.*, 2005; Campbell and Sage, 2006). If CO_2_ concentration does influence seed size evolution, then plants of different photosynthetic pathways should also differ in seed size. C_4_ plants concentrate CO_2_ around Rubisco and suppress photorespiration, such that they typically outperform C_3_ plants in low CO_2_ environments (Dippery *et al.*, 1995; Tissue *et al.*, 1995; Cunniff *et al.*, 2017). In recent atmospheres, C_4_ grasses exhibit greater seedling growth rates and resource use efficiencies than C_3_ grasses of equivalent seed size, allowing faster establishment (Simpson *et al.*, 2021). Consistently, C_4_ grasses produce smaller seeds than C_3_ grasses (Csontos and Kalapos, 2013). Smaller seed sizes of C_4_ plants could also indicate how seed size might change in C_3_ plants adapting to high CO_2_ atmospheres.

One implication of our results is that rising CO_2_ may offset the advantages conferred by larger seeds; in elevated atmospheric CO_2_, small-seeded plants could exhibit greater photosynthesis, growth and establishment shortly after germination than they would in low CO_2_ (Campbell and Sage, 2005; Gerhardt and Ward, 2010). As a consequence, the cost of having small seeds would be reduced, while the benefits of greater fecundity and colonization in small-seeded plants would be more apparent. Based on this reasoning, we hypothesize there may be an evolutionary shift towards production of greater numbers of smaller seeds in future, high CO_2_ environments, all things being equal. This hypothesis is not clearly supported by the current literature, however. Rising CO_2_ is observed to favour greater productivity gains in large- than in small-seeded species of similar growth form by simply allowing the larger seedlings to grow faster in an absolute sense than smaller seedlings (Steinger *et al.*, 2000; Huxman *et al.*, 2001; Jones and Reekie, 2007; Zhang and Granger, 2014; Poorter *et al.*, 2022). These results suggest that the greater provisioning potential of higher CO_2_, coupled with faster initial growth, could favour evolution of larger seeds in CO_2_-enriched atmospheres, in contrast to our hypothesis. We argue that such a conclusion is premature. Elevated CO_2_ trials to date emphasize single-generation studies of plant responses to CO_2_, and typically examined plants grown in isolation from community interactions such as competition through which fecundity differences become important (Ward and Kelly, 2004; Lau *et al.*, 2014). Fitness advantages of greater seed size vs. number under elevated CO_2_ should be evaluated using multi-generational studies that allow for consequences of seed number variation to be realized in a realistic community context (Turnbull *et al.*, 1999; Lau *et al.*, 2014). With respect to seed evolution, Ward *et al.* (2000) noted increases in seed number in *Arabidopsis thaliana* after five generations when genotypes with higher seed number were selected for inclusion in the next generation at both 180 and 700 ppm growth CO_2_. Our study also suffers from being a single-generation study examining plants in isolation; however, unlike elevated CO_2_ studies where the limits of survival and productivity are generally not approached, our results demonstrate that the combination of low CO_2_ and warmth creates situations where seedlings fail in a differential pattern that relates to seed size and CO_2_ deficiency. This failure indicates the possibility of strong selection pressure against small-seeded plants in low CO_2_ conditions.

Finally, one potentially important implication of low CO_2_ effects on seed size evolution are the consequences for humanity. By favouring larger seed size, low CO_2_ conditions of the past few million years may have facilitated the origin of agriculture. Larger seeds would have been more likely to attract the attention of proto-farmers early in the domestication process (Purugganan and Fuller, 2009), and clades such as legumes (beans, peas and other pulses) and the BEP clade of cultivated grasses (wheat, rice, barley and other grains) are notable for the repeated domestications of relatively large-seeded C_3_ crops (Harlan, 1992). If low CO_2_ conditions had not occurred in Earth’s recent history, then it is possible the Earth’s flora may not have been as suitable for domestication, such that agriculture, and the civilization it supports, may not have occurred. By considering such possibilities, it is possible for plant biologists to gain new insights into how past environments influenced the modern world, and how the future world may respond to climate and atmospheric change.

## SUPPLEMENTARY DATA

Supplementary data are available online at https://academic.oup.com/aob and consist of the following. Table S1: name, source and seed mass for the bean genotypes used in this study. Table S2: three-way ANOVA table for CO_2_, growth temperature and seed mass effects on leaf area or shoot mass at 14 DAP in five bean genotypes shown in Figs 2 and S2. Figure S1: atmospheric CO_2_ concentrations estimated for the past 40 million years and the past 800 000 years. Figure S2: shoot dry mass at 14 DAP for five bean varieties grown at three ambient CO_2_ concentrations at day/night temperatures of either 25 °C/19 °C C, 30 °C/24 °C or 35 °C/29 °C. Figure S3: growth temperature effect on the percentage reduction in leaf area and shoot mass for large- and small-seeded bean genotypes at low growth CO_2_ concentration relative to 370 ppm growth CO_2_.

mcac112_suppl_Supplementary_Material
