# Supplementary material for: Seed size effects on plant establishment under low atmospheric CO2, with implications for seed size evolution
Source: Ann Bot. 2022 Sep 12;130(6):825–34. doi: 10.1093/aob/mcac112 (PMC9758303; doi:10.1093/aob/mcac112)
Supplement: mcac112_suppl_Supplementary_Material [file mcac112_suppl_supplementary_material.pptx]

## Slide 1
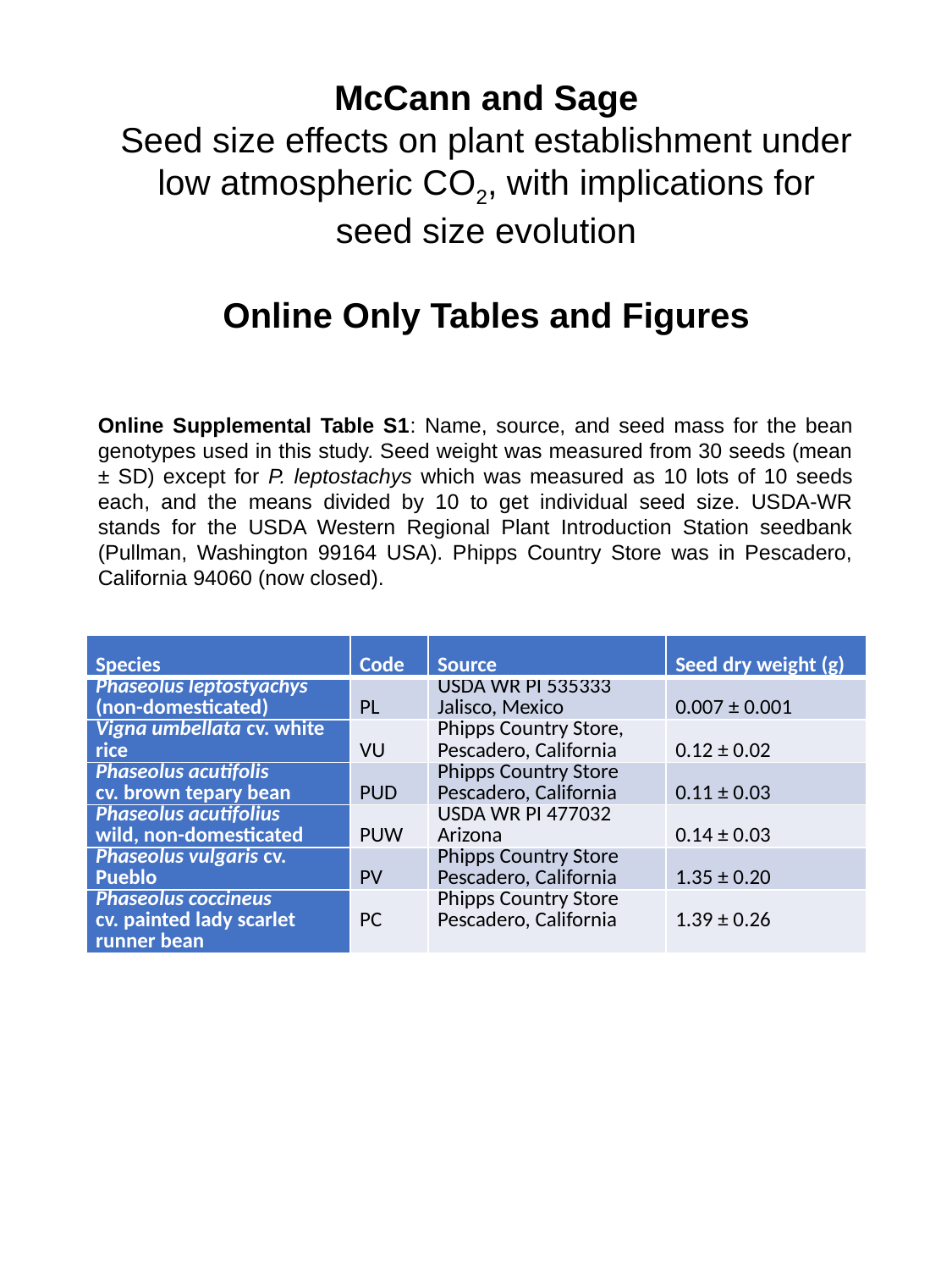

McCann and Sage
Seed size effects on plant establishment under low atmospheric CO2, with implications for seed size evolution
Online Only Tables and Figures
Online Supplemental Table S1: Name, source, and seed mass for the bean genotypes used in this study. Seed weight was measured from 30 seeds (mean ± SD) except for P. leptostachys which was measured as 10 lots of 10 seeds each, and the means divided by 10 to get individual seed size. USDA-WR stands for the USDA Western Regional Plant Introduction Station seedbank (Pullman, Washington 99164 USA). Phipps Country Store was in Pescadero, California 94060 (now closed).
| Species | Code | Source | Seed dry weight (g) |
| --- | --- | --- | --- |
| Phaseolus leptostyachys (non-domesticated) | PL | USDA WR PI 535333 Jalisco, Mexico | 0.007 ± 0.001 |
| Vigna umbellata cv. white rice | VU | Phipps Country Store, Pescadero, California | 0.12 ± 0.02 |
| Phaseolus acutifolis cv. brown tepary bean | PUD | Phipps Country Store Pescadero, California | 0.11 ± 0.03 |
| Phaseolus acutifolius wild, non-domesticated | PUW | USDA WR PI 477032 Arizona | 0.14 ± 0.03 |
| Phaseolus vulgaris cv. Pueblo | PV | Phipps Country Store Pescadero, California | 1.35 ± 0.20 |
| Phaseolus coccineus cv. painted lady scarlet runner bean | PC | Phipps Country Store Pescadero, California | 1.39 ± 0.26 |

## Slide 2
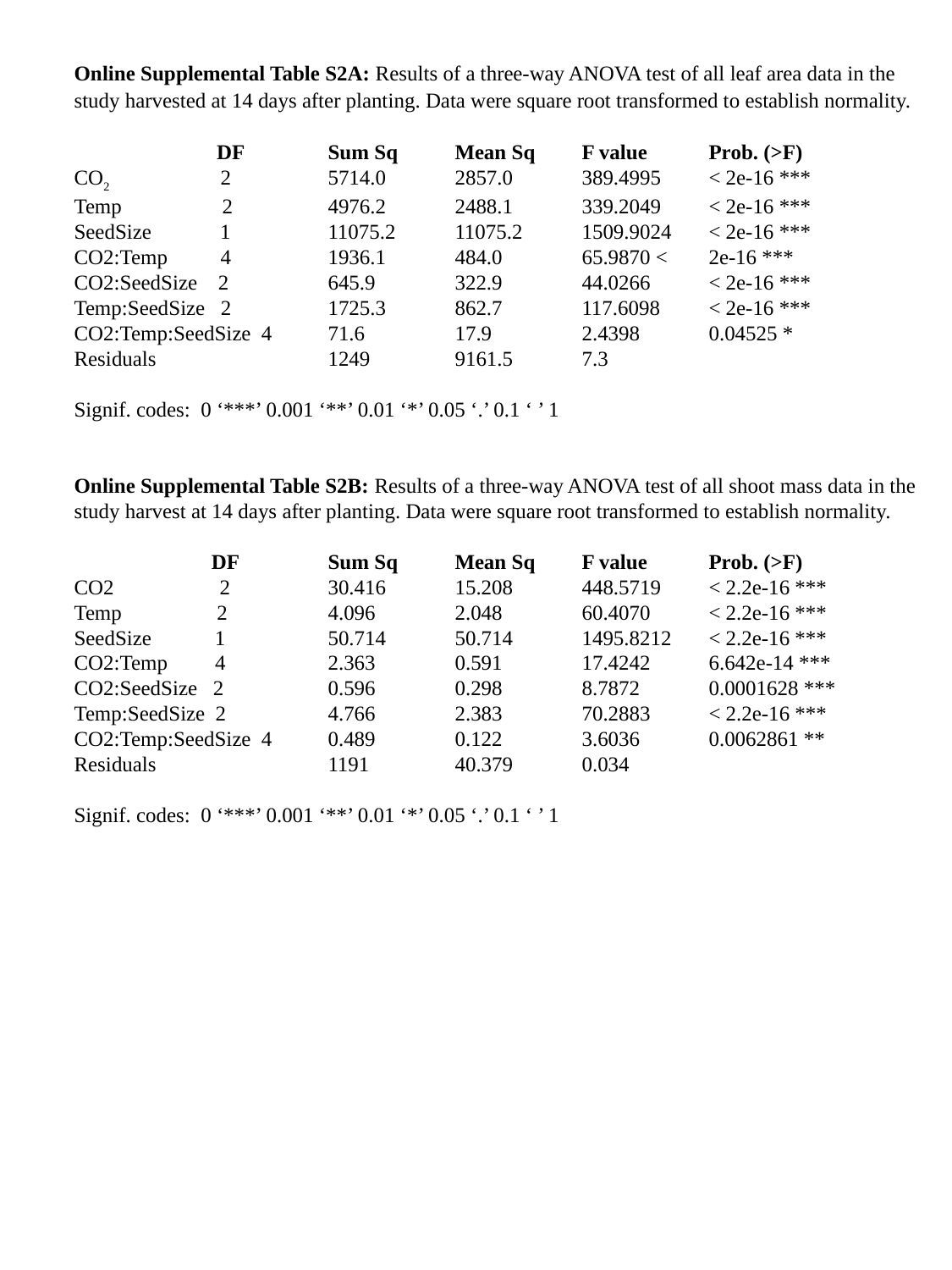

Online Supplemental Table S2A: Results of a three-way ANOVA test of all leaf area data in the study harvested at 14 days after planting. Data were square root transformed to establish normality.
                 	 DF   	Sum Sq 	Mean Sq   	F value  	Prob. (>F)    
CO2                   2	5714.0  	2857.0  	389.4995 	< 2e-16 ***
Temp                 2  	4976.2  	2488.1  	339.2049 	< 2e-16 ***
SeedSize           1 	11075.2 	11075.2 	1509.9024 	< 2e-16 ***
CO2:Temp       4  	1936.1   	484.0   	65.9870 < 	2e-16 ***
CO2:SeedSize 2   	645.9   	322.9   	44.0266 	< 2e-16 ***
Temp:SeedSize   2  	1725.3   	862.7  	117.6098 	< 2e-16 ***
CO2:Temp:SeedSize  4    	71.6    	17.9    	2.4398 	0.04525 *  
Residuals         	1249  	9161.5     	7.3
Signif. codes:  0 ‘***’ 0.001 ‘**’ 0.01 ‘*’ 0.05 ‘.’ 0.1 ‘ ’ 1
Online Supplemental Table S2B: Results of a three-way ANOVA test of all shoot mass data in the study harvest at 14 days after planting. Data were square root transformed to establish normality.
                    	 DF	Sum Sq 	Mean Sq   	F value    	Prob. (>F)    
CO2                2 	30.416  	15.208  	448.5719 	< 2.2e-16 ***
Temp               2  	4.096   	2.048   	60.4070 	< 2.2e-16 ***
SeedSize         1 	50.714  	50.714 	1495.8212 	< 2.2e-16 ***
CO2:Temp     4  	2.363   	0.591   	17.4242 	6.642e-14 ***
CO2:SeedSize 2  	0.596   	0.298    	8.7872 	0.0001628 ***
Temp:SeedSize  2  	4.766   	2.383   	70.2883 	< 2.2e-16 ***
CO2:Temp:SeedSize  4  	0.489   	0.122    	3.6036 	0.0062861 **
Residuals    		1191 	40.379   	0.034                        
Signif. codes:  0 ‘***’ 0.001 ‘**’ 0.01 ‘*’ 0.05 ‘.’ 0.1 ‘ ’ 1

## Slide 3
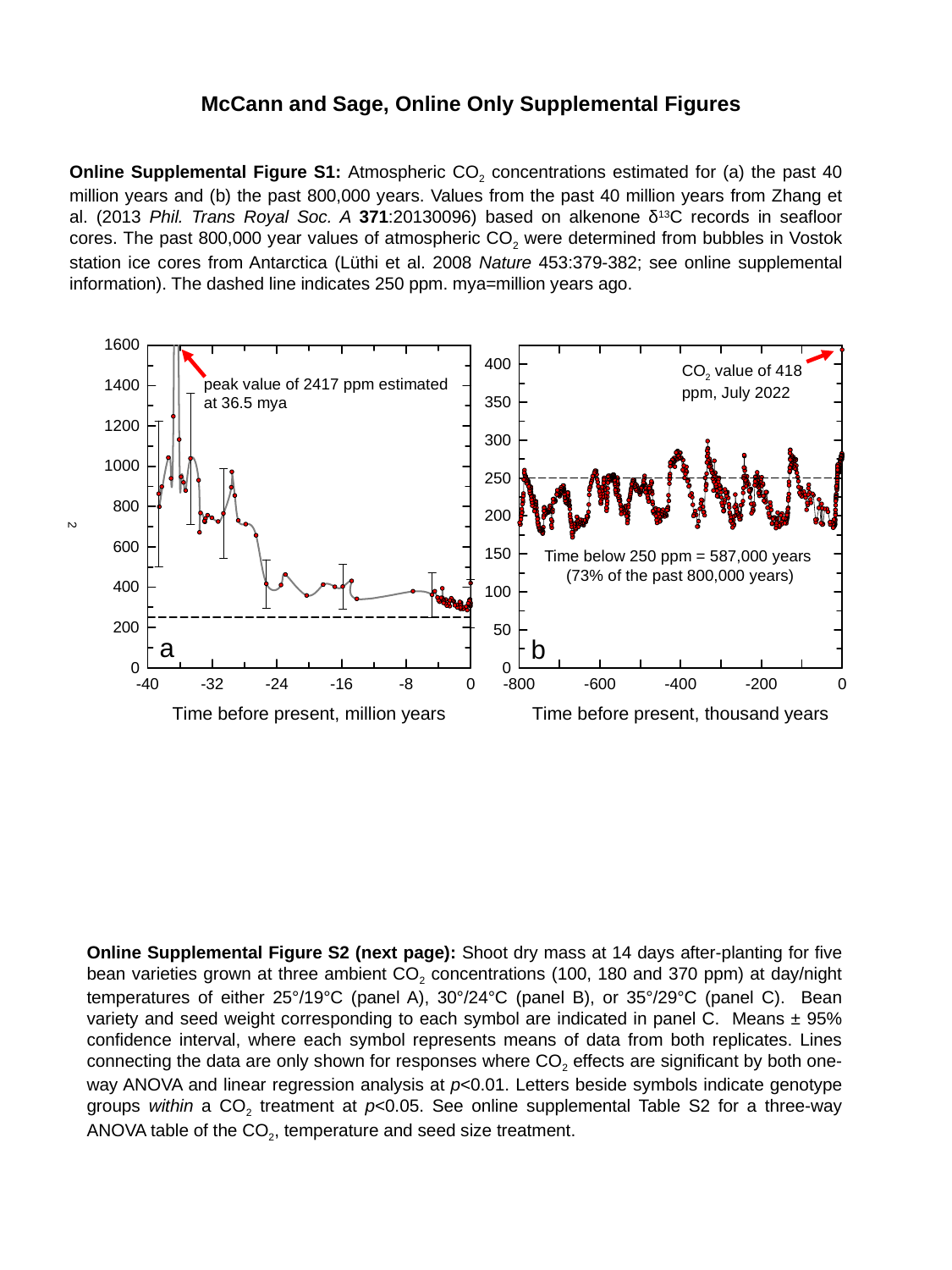

McCann and Sage, Online Only Supplemental Figures
Online Supplemental Figure S1: Atmospheric CO2 concentrations estimated for (a) the past 40 million years and (b) the past 800,000 years. Values from the past 40 million years from Zhang et al. (2013 Phil. Trans Royal Soc. A 371:20130096) based on alkenone δ13C records in seafloor cores. The past 800,000 year values of atmospheric CO2 were determined from bubbles in Vostok station ice cores from Antarctica (Lüthi et al. 2008 Nature 453:379-382; see online supplemental information). The dashed line indicates 250 ppm. mya=million years ago.
CO2 value of 418
ppm, July 2022
peak value of 2417 ppm estimated
at 36.5 mya
Time below 250 ppm = 587,000 years
(73% of the past 800,000 years)
a
b
Online Supplemental Figure S2 (next page): Shoot dry mass at 14 days after-planting for five bean varieties grown at three ambient CO2 concentrations (100, 180 and 370 ppm) at day/night temperatures of either 25°/19°C (panel A), 30°/24°C (panel B), or 35°/29°C (panel C). Bean variety and seed weight corresponding to each symbol are indicated in panel C. Means ± 95% confidence interval, where each symbol represents means of data from both replicates. Lines connecting the data are only shown for responses where CO2 effects are significant by both one-way ANOVA and linear regression analysis at p<0.01. Letters beside symbols indicate genotype groups within a CO2 treatment at p<0.05. See online supplemental Table S2 for a three-way ANOVA table of the CO2, temperature and seed size treatment.

## Slide 4
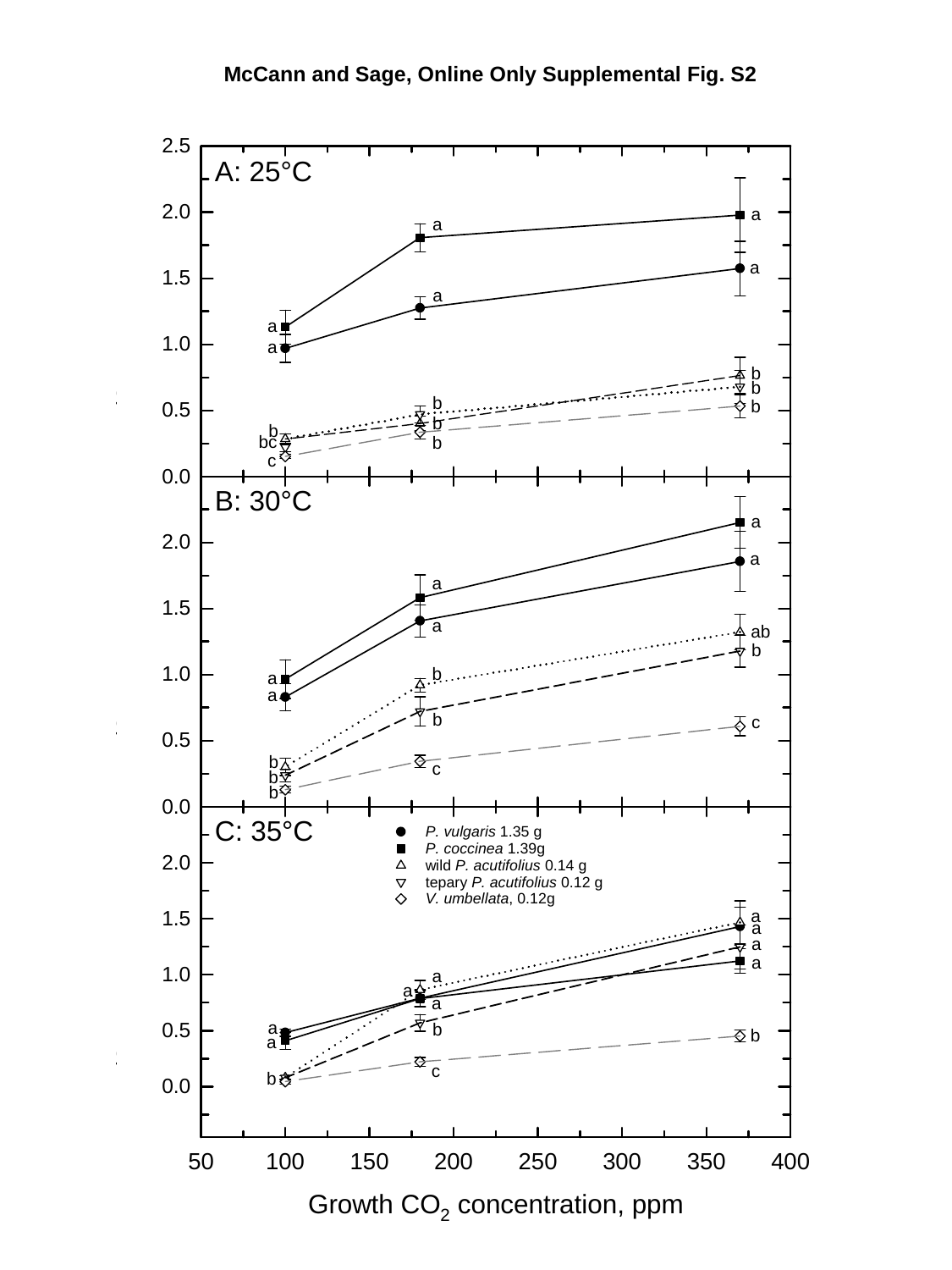

McCann and Sage, Online Only Supplemental Fig. S2
McCann and Sage, Fig. 3
A: 25°C
a
a
a
a
a
a
b
b
b
b
b
b
bc
b
c
B: 30°C
a
a
a
a
ab
b
b
a
a
b
c
b
c
b
b
C: 35°C
a
a
a
a
a
a
a
a
b
b
a
c
b
a
b
b
a
c
b

## Slide 5
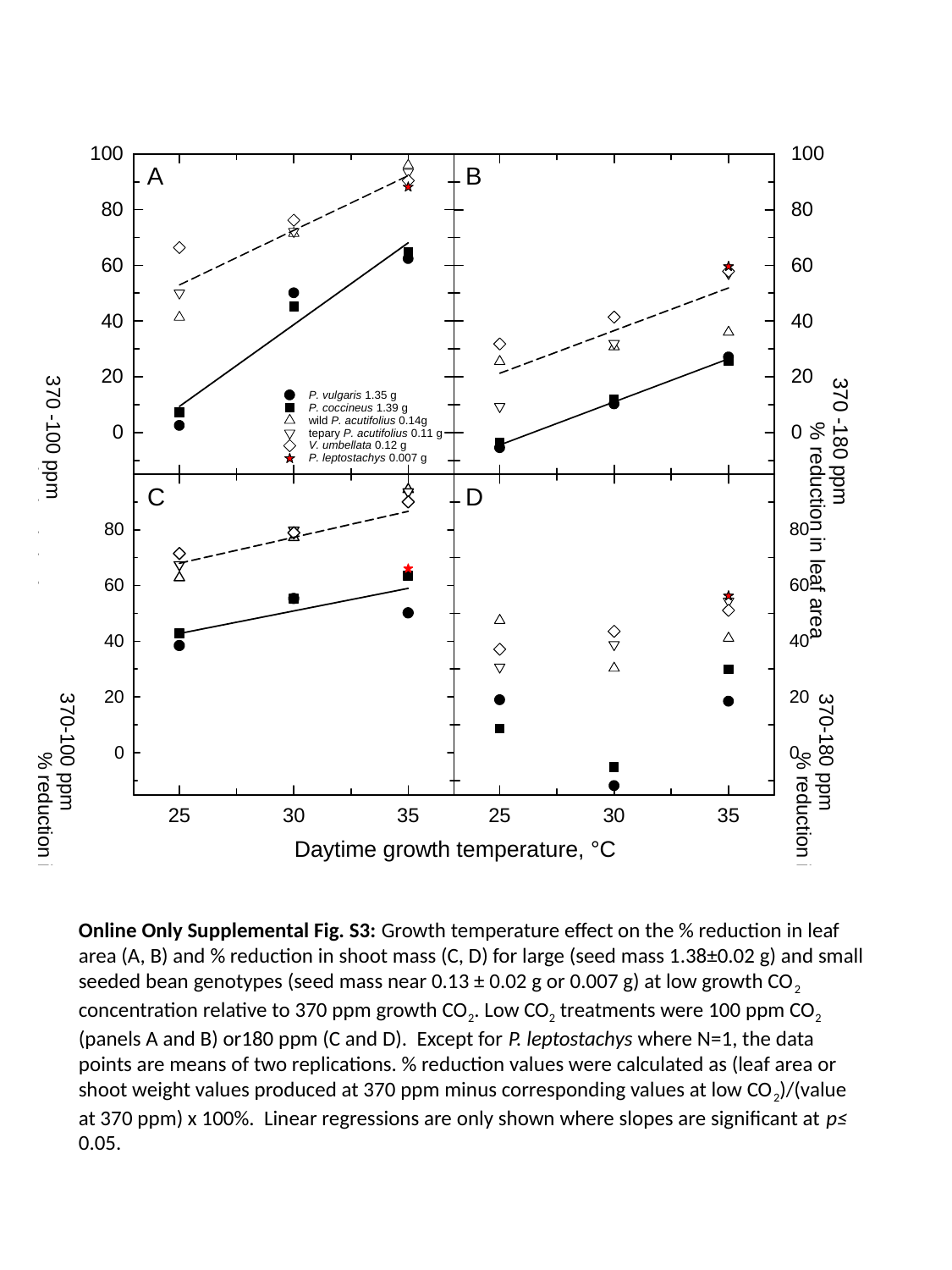

A
B
D
C
Online Only Supplemental Fig. S3: Growth temperature effect on the % reduction in leaf area (A, B) and % reduction in shoot mass (C, D) for large (seed mass 1.38±0.02 g) and small seeded bean genotypes (seed mass near 0.13 ± 0.02 g or 0.007 g) at low growth CO2 concentration relative to 370 ppm growth CO2. Low CO2 treatments were 100 ppm CO2 (panels A and B) or180 ppm (C and D). Except for P. leptostachys where N=1, the data points are means of two replications. % reduction values were calculated as (leaf area or shoot weight values produced at 370 ppm minus corresponding values at low CO2)/(value at 370 ppm) x 100%. Linear regressions are only shown where slopes are significant at p≤ 0.05.
